# Ultra-processed food consumption, hedonic hunger, and sleep quality among university students: a food and nutrition literacy perspective

**DOI:** 10.3389/fpubh.2026.1785585

**Published:** 2026-02-16

**Authors:** Sabriye Arslan, Meryem Saban Güler, Asude Beyza Köse, İlayda Kaygusuz, İremnaz Demir, Sena Delioǧlu

**Affiliations:** 1 Department of Nutrition and Dietetics, Faculty of Health Sciences, Gazi University, Ankara, Türkiye; 2 Department of Nutrition and Dietetics, Faculty of Health Sciences, Batman University, Batman, Türkiye

**Keywords:** emotional eating, food and nutrition literacy, hedonic hunger, sleep quality, ultra-processed foods

## Abstract

**Background:**

Ultra-processed foods (UPFs) pose a significant health risk for university students during a critical transitional stage of life. This study aimed to investigate the effects of food and nutrition literacy on UPFs consumption, hedonic hunger, and sleep quality among university students.

**Methods:**

This study was conducted with 1,400 university students. Sociodemographic characteristics, health status, dietary habits, and anthropometric measurements were collected through face-to-face interviews using a structured questionnaire administered by the researchers. Participants also completed the Food and Nutrition Literacy Instrument (FNL), the Screening Questionnaire for Highly Processed Food Consumption (sQ-HPF), the Power of Food Scale (PFS), Emotional Eater Questionnaire (EEQ) and the Pittsburgh Sleep Quality Index (PSQI). The data obtained from the study were analyzed using SPSS version 23.0.

**Results:**

Females had significantly higher scores than males in the knowledge and attitude sub-dimensions of FNL (*p* < 0.001), whereas no gender-related difference was observed in the behavior sub-dimension. Females also exhibited significantly higher PFS and EEQ scores compared with males (*p* < 0.05), and poorer sleep quality as indicated by higher PSQI scores (*p* < 0.01). No significant gender differences were found in UPF consumption. When FNL levels were examined, individuals with lower literacy levels demonstrated higher hedonic hunger, poorer sleep quality, greater UPF consumption, and more pronounced emotional eating behaviors (*p* < 0.05). In linear regression analyses, the knowledge and attitude sub-dimensions of FNL were negatively associated with age, gender, emotional eating, and UPFs consumption, and positively associated with hedonic hunger (*p* < 0.001).

**Conclusions:**

This study demonstrates that food and nutrition literacy among university students is significantly associated with hedonic hunger, emotional eating, sleep quality, and UPFs consumption. These findings suggest that nutrition interventions targeting young adults should extend beyond knowledge transfer and incorporate behavioral and psychosocial components to promote healthier eating patterns and overall well-being.

## 1 Introduction

The global food system has experienced a significant transition in recent years due to industrialization, urbanization, and technological improvements (1). The most prominent change associated with this transformation is the shift from natural and minimally processed foods to a dietary pattern based on highly processed, industrial formulations (2–4). A food classification system called NOVA has been designed to enhance the understanding of the increasingly complexity structure of produced foods, their evolving composition and palatability, and to investigate the impact of processing on food quality and health (5). NOVA has been acknowledged as a thorough framework for assessing the degrees of food processing. The NOVA system classifies foods into four categories according to the nature, extent, and intent of the industrial processing utilized in their production. At the highest level of the processing spectrum ultra-processed foods (UPFs) are defined as industrial compositions including several ingredients subjected to extensive processing (2, 5, 6). UPFs constitute a substantial proportion of daily dietary energy intake in modern societies and are widely consumed, particularly among young adults. University students represent a high-risk group for UPF consumption due to being in a critical transitional period of life, beginning to make independent food choices, and facing factors such as irregular lifestyles and time constraints (7, 8). In this period, increased intake of UPFs relates not only with personal preferences but also with various criteria like gender, age, socioeconomic status, level of education, and urban living conditions (4, 9, 10). Among these factors, food and nutrition literacy (FNL), which reflects an individual's capacity to accurately evaluate the food environment and make healthy choices, has been gaining increasing importance in determining UPF consumption. FNL refers to the capacity of individuals to access nutrition-related information, understand and evaluate this information, and make healthy dietary decisions (11, 12). Beyond being a simple accumulation of knowledge, this concept encompasses functional, interactive, and critical skills, including the ability to analyze food labels, interpret nutrient declarations, apply healthy cooking techniques, and develop a critical stance toward food marketing strategies (11, 13).

In today's complex food environment, FNL which represents the cognitive and practical competencies required for individuals to make healthy food choices emerges as an important protective mechanism against UPF consumption, particularly for groups such as university students who are exposed to intense marketing stimuli and operate within fast-paced consumption environments (13, 14).

Although the consequences of UPF consumption on physical health have been extensively addressed, the effects of chronic UPF intake on mental health have been examined in a relatively more limited manner (15–17). UPFs affect biological processes, such as dopamine signaling, possibly leading to impaired psychological performance in certain individuals (18). Studies demonstrate that individuals who are prone to hedonic hunger exhibit increased consumption of fatty meals, sweets, and unhealthy snacks, a trend correlated with psychological states like depressive symptoms, stress, and anxiety (17, 19). In addition, impairments in sleep duration and quality are known to stimulate appetite and food intake (20). Individuals experiencing sleep deprivation become more sensitive to rewarding food cues; this condition is thought to enhance the hedonic component of food intake, thereby triggering increased energy consumption and potentially playing a role in the pathophysiology of obesity. It has been emphasized that acute sleep deprivation can increase the desire to consume food by enhancing hedonic stimulation, independently of plasma glucose levels (21, 22). UPF consumption has been reported as a factor influencing both the onset of insomnia and sleep quality (22).

The aim of this study is to examine the effects of FNL levels of university students on UPF consumption, hedonic hunger, emotional eating behaviors, and sleep status.

## 2 Materials and methods

### 2.1 Study design and sample

This descriptive cross-sectional study was conducted between 1 March 2025 and 1 September 2025. Within the scope of the study, 1,562 individuals were reached. However, a total of 162 individuals were excluded from the study due to 112 participants declining to complete the questionnaire, 11 participants being vegan/vegetarian, and 39 participants having chronic or psychiatric diseases. Consequently, the study included a total of 1,400 university students, comprising 522 males and 878 females. Data were collected through face-to-face interviews using a structured questionnaire administered by the researchers. Participants were recruited using a snowball sampling method from universities located in Ankara, Türkiye.

Individuals were eligible for inclusion if they were enrolled as university students, and voluntarily agreed to participate in the study after being informed about the research objectives and procedures. Exclusion criteria included being under 18 years of age, having a self-reported diagnosis of a chronic disease or psychiatric disorder, following a vegan or vegetarian diet, having applied a special weight-loss diet within the past year, or declining to participate in the study. All exclusion criteria were determined based on participants' self-reports. The questionnaire collected information on sociodemographic characteristics, dietary habits, food consumption, anthropometric measurements, sleep quality, FNL, hedonic hunger, emotional eating, and UPF consumption. Anthropometric measurements and questionnaire data were obtained during face-to-face interviews conducted by trained researchers. On average, completion of the questionnaire took approximately 15–20 min.

Ethical approval for the study was obtained from the Gazi University Scientific Research Ethics Committee (Decision No: 2025-25, Date: 24.12.2024). All participants provided written informed consent prior to participation, and the study was conducted in accordance with the principles of the Declaration of Helsinki.

### 2.2 Anthropometric measurements

Body weight and height information of the participants were collected using a self-reported method. Participants' body weight status was evaluated using body mass index (BMI) according to the classification recommended by the World Health Organization (WHO). BMI was calculated by dividing body weight (kg) by the square of height in meters (m^2^) (kg/m^2^). According to the WHO classification, a BMI of < 18.5 kg/m^2^ is classified as underweight, 18.5–24.9 kg/m^2^ as normal weight, 25.0–29.9 kg/m^2^ as overweight, and ≥30.0 kg/m^2^ as obesity (23).

### 2.3 Food and nutrition literacy (FNL)

Food and nutrition literacy was assessed using the Food and Nutrition Literacy (FNL) instrument, developed by Demir and Özer (24). The FNL is a 36-item self-report measure comprising three sub-dimensions: knowledge (13 items), attitude (13 items), and behavior (10 items). The instrument includes both knowledge-based questions and Likert-type statements, with sub-dimensional scores calculated independently. The ranges of total scores between the minimum and maximum values were 0–13 points for the knowledge domain, 13–65 points for the attitude domain, and 10–50 points for the behavior domain. Sub-dimensional scores were categorized as excellent, limited, or inadequate based on established cut-off values. For the knowledge domain, scores of 0–9 were classified as inadequate, 10–11 as limited, and 12–13 as excellent; the corresponding cut-offs were 0–43, 44–51, and 52–65 for the attitude domain, and 0–25, 26–33, and 34–50 for the behavior domain. In the original validation study, Cronbach's alpha coefficients were reported as 0.605 for the knowledge domain, 0.761 for the attitude domain, and 0.727 for the behavior domain (24).

### 2.4 Power of food scale (PFS)

To assess the effect of hedonic hunger on food choice, the Turkish version of the Power of Food Scale (PFS-Tr) was used as a self-report measure. The scale was originally developed by Lowe et al., (25). and its Turkish validity and reliability were established by Ülker et al. (26). It consists of 13 items rated on a five-point Likert scale (1 = strongly disagree to 5 = strongly agree) and comprises three subscales: food available, which assesses general thoughts about foods; food present, which evaluates attraction to food that is directly accessible to the individual; and food tasted, which measures desire for and pleasure derived from food upon initial tasting. Total and subscale scores are calculated by summing the item scores and dividing by the total number of items, yielding scores ranging from 1 to 5. Higher scores indicate greater responsiveness to the food environment and a higher tendency toward hedonic hunger (26).

### 2.5 Pittsburgh sleep quality index (PSQI)

Sleep quality was assessed using the Pittsburgh Sleep Quality Index (PSQI), originally developed by Buysse et al. (27). The Turkish adaptation of the scale, including its validity and reliability studies, was conducted by Agargün et al. The PSQI evaluates sleep quality and sleep disturbances over the previous month. It consists of 24 items, of which 18 contribute to scoring and are grouped into seven components: subjective sleep quality, sleep latency, sleep duration, habitual sleep efficiency, sleep disturbances, use of sleep medication, and daytime dysfunction. Each component is scored on a 0–3 scale, with higher scores indicating greater sleep-related difficulties. The sum of the seven component scores yields a global PSQI score ranging from 0 to 21. A total score of five or lower indicates good sleep quality, whereas a score above five indicates poor sleep quality. The Turkish version of the PSQI has demonstrated good internal consistency, with a Cronbach's alpha coefficient of 0.80 (28).

### 2.6 Ultra-Processed foods consumption

UPF consumption was assessed using the Screening Questionnaire for Highly Processed Food Consumption (sQ-HPF). The questionnaire was originally developed by Martínez-Pérez et al. to provide a practical assessment of highly processed food consumption (29). The Turkish adaptation and validation of the instrument were conducted by Erdogan Gövez et al. (30). The Turkish version of the sQ-HPF consists of 11 items, each assessing the consumption of specific highly processed food products. Participants respond with “yes” or “no” for each item, with one point assigned for affirmative responses and 0 points for negative responses, yielding a total score ranging from 0 to 11. Higher total scores indicate greater consumption of UPFs. Based on established cut-off values, individuals with a total score of six or higher are classified as having high UPF consumption. The internal consistency of the Turkish version of the scale was acceptable, with a Cronbach's alpha coefficient of 0.65 (30).

### 2.7 Emotional eater questionnaire

Emotional eating was assessed using the Emotional Eater Questionnaire (EEQ), originally developed by Garaulet et al. (58) to evaluate emotional eating behaviors. The Turkish adaptation, validity, and reliability of the scale were conducted by Arslantaş et al. (31). The EEQ consists of 10 items and three subscales: disinhibition (loss of control overeating), type of food (preference for specific foods, particularly sweets), and guilt (feelings of guilt following food consumption). Responses are rated on a four-point Likert scale ranging from 0 (never) to 3 (always). Total scores range from 0 to 30, with higher scores indicating a greater level of emotional eating behavior. The internal consistency of the Turkish version of the EEQ was good, with a Cronbach's alpha coefficient of 0.84. Subscale reliability coefficients were reported as 0.81 for disinhibition, 0.57 for type of food, and 0.64 for guilt (31).

### 2.8 Statistical analysis

*Post-hoc* power analysis was performed using G^*^Power (version 3.1.9.7, Universitat Düsseldorf, Düsseldorf, Germany). Based on the observed Spearman correlation between the attitude sub-dimension of FNL and UPF consumption (*r* = −0.172), assuming a two-tailed alpha level of 0.05 and a total sample size of 1,400 participants, the achieved statistical power (1–β) exceeded 99%. The data obtained from the study were analyzed and visualized using SPSS 23.0 (Statistical Package for Social Sciences, Inc., Chicago, IL, USA). The normality of continuous variables was assessed using skewness and kurtosis values as well as graphical methods. As the distributions were consistent with parametric assumptions, parametric statistical tests were applied. Descriptive statistics were presented as mean ± standard deviation (SD) for continuous variables and as number (percentage) for categorical variables. Comparisons of continuous variables between males and females were performed using the independent samples *t*-test, while Chi-square tests were used for comparisons of categorical variables. Differences in mean scores of hedonic hunger, sleep quality, UPF consumption, and emotional eating across FNL levels (inadequate, limited, and excellent) were evaluated using one-way ANOVA. To control for Type I error inflation due to multiple comparisons, *post hoc* tests were applied to identify pairwise group differences. Levene's test was used to assess the homogeneity of variances; Tukey's HSD test was applied as a multiple comparison adjustment when variances were homogeneous, and the Games–Howell test was used to adjust for multiple testing when this assumption was not met.

Associations between age, FNL sub-dimensions, UPF, hedonic hunger, sleep quality, emotional eating, and anthropometric variables were examined using Spearman's rank correlation analysis, considering the ordinal structure of some scale scores. To identify factors independently associated with FNL sub-dimensions, multiple linear regression analyses were conducted. Separate regression models were constructed for each sub-dimension as the dependent variable. Age, gender, body mass index (BMI), hedonic hunger (PFS total score), sleep quality (PSQI total score), UPF consumption (sQ-HPF total score), and emotional eating (EEQ total score) were entered simultaneously into the models using the enter method, based on theoretical relevance and previous literature rather than solely on bivariate associations. Multicollinearity was assessed using collinearity diagnostics. All remaining assumptions for multiple linear regression analyses, including linearity, normality and independence of residuals, and homoscedasticity, were evaluated and met. Statistical significance was considered at *p* < 0.05 within a 95% confidence interval.

## 3 Results

The general characteristics of the participants are presented in Table 1. Of the 1,400 university students included in the study, 62.7% were female and 37.3% were male. The mean age of the participants was 22.1 ± 1.88 years, and the mean body mass index (BMI) was 23.4 ± 3.58 kg/m^2^. According to BMI classification, the majority of participants (67.1%) had normal body weight. In addition, 5.1% of the participants were underweight, 22.9% were classified as overweight, and 4.8% were classified as obese. Dietary supplement use was reported by 16.7% of the participants, whereas the majority (83.3%) did not report using any supplements. Regarding meal patterns, most participants reported consuming two or three main meals per day. Overall, 57.6% consumed two main meals and 38.9% consumed three main meals daily. Snack consumption was also prevalent, with 46.4% of participants reporting one snack per day and 24.0% reporting two snacks per day. Only a small proportion of participants (21.7%) reported no snack consumption.

**Table 1:** Sociodemographic, anthropometric, and lifestyle characteristics of the study population by gender.

**Characteristics**	**Males (*n* = 522) Mean ±SD or *n* (%)**	**Females (*n* = 878) Mean ±SD or *n* (%)**	**Total (*n* = 1,400) Mean ±SD or *n* (%)**
Age (y)	22.2 ± 2.07	22.0 ± 1.76	22.1 ± 1.88
Body weight (kg)	77.6 ± 10.72	60.6 ± 10.98	66.9 ± 13.62
Height (cm)	177.8 ± 6.38	163.0 ± 5.42	168.5 ± 9.22
BMI (kg/m^2^)	24.5 ± 2.86	22.8 ± 3.80	23.4 ± 3.58
**BMI classification**			
Underweight	6 (1.1)	66 (7.5)	72 (5.1)
Normal	330 (63.2)	610 (69.5)	940 (67.1)
Overweight	161 (30.8)	160 (18.2)	321 (22.9)
Obese	25 (4.8)	42 (4.8)	67 (4.8)
**Dietary supplement use**			
Yes	60 (11.5)	174 (19.8)	234 (16.7)
No	462 (88.5)	704 (80.2)	1,166 (83.3)
**Number of meals**			
**Main meals**			
1	8 (1.5)	41 (4.7)	49 (3.5)
2	262 (50.2)	545 (62.1)	807 (57.6)
3	252 (48.3)	292 (33.3)	544 (38.9)
**Snacks**			
0	149 (28.5)	155 (17.7)	304 (21.7)
1	234 (44.8)	415 (47.3)	649 (46.4)
2	102 (19.5)	234 (26.7)	336 (24.0)
3	37 (7.1)	74 (8.4)	111 (7.9)

All percentages are calculated in columns.

BMI, Body Mass Index.

Table 2 summarizes the distribution and mean scores of FNL sub-dimensions and related assessment tools according to gender. Statistically significant gender differences were observed in both the total scores and categorical distributions of the knowledge and attitude sub-dimensions of FNL (*p* < 0.001 for both). In the knowledge sub-dimension, 26.0% of females were classified in the excellent category, compared with 13.6% of males. Similarly, for the attitude sub-dimension, 22.9% of females were classified as having an excellent level, whereas this proportion was 14.4% among males (both *p* < 0.001). In contrast, no significant gender differences were observed in either the categorical distribution or the total scores of the behavior sub-dimension (*p* > 0.05). With regard to hedonic hunger, females demonstrated significantly higher total scores on the Power of Food Scale (PFS) compared to males (*p* = 0.020). Subscale analyses revealed that females had significantly higher scores on the food present (*p* = 0.034) and food taste (*p* = 0.015) subscales, while no significant difference was observed for the food available subscale (*p* > 0.05). Sleep quality also differed significantly by gender. Females had higher PSQI total scores, indicating poorer sleep quality compared with males (*p* = 0.007). Accordingly, the prevalence of poor sleep quality was significantly higher among females (80.6%) than males (72.8%), whereas good sleep quality was more frequently observed in males (27.2%) compared with females (19.4%) (*p* = 0.001). No statistically significant gender differences were found in UPF consumption, either in terms of total sQ-HPF scores or low–high consumption categories (*p* > 0.05). However, emotional eating differed significantly by gender, with females exhibiting higher total scores on the Emotional Eater Questionnaire (EEQ) compared to males (*p* = 0.019). Among EEQ sub-dimensions, guilt scores were significantly higher in females (*p* < 0.001), whereas no significant gender differences were observed for disinhibition or type of food subscales (*p* > 0.05).

**Table 2:** Distribution and scores of assessment tools according to gender.

**Variables**	**Males (*n* = 522) Mean ±SD or *n* (%)**	**Females (*n* = 878) Mean ±SD or *n* (%)**	**Total (*n* = 1,400) Mean ±SD or *n* (%)**	***p***
**FNL sub-dimensions**				
**Knowledge**				
Inadequate	324 (62.1)	373 (42.5)	697 (49.8)	< 0.001#
Limited	127 (24.3)	277 (31.5)	404 (28.9)	
Excellent	71 (13.6)	228 (26.0)	299 (21.4)	
Knowledge total scores	7.7 ± 3.22	9.2 ± 2.63	8.7 ± 2.95	< 0.001^§^
**Attitude**				
Inadequate	295 (56.5)	295 (33.6)	590 (42.1)	< 0.001#
Limited	152 (29.1)	382 (43.5)	534 (38.1)	
Excellent	75 (14.4)	201 (22.9)	276 (19.7)	
Attitude total scores	42.0 ± 8.97	46.3 ± 7.10	44.7 ± 8.12	< 0.001^§^
**Behavior**				
Inadequate	138 (26.4)	200 (22.8)	338 (24.1)	0.097#
Limited	203 (38.9)	391 (44.5)	594 (42.4)	
Excellent	181 (34.7)	287 (32.7)	468 (33.4)	
Behavior total scores	30.4 ± 7.31	30.3 ± 7.15	30.4 ± 7.21	0.951^§^
**PFS total score**	**3.2** **±0.80**	**3.3** **±0.82**	**3.3** **±0.82**	**0.020** ^**§**^
**PFS subscales**				
Food available	3.0 ± 0.93	3.1 ± 0.98	3.0 ± 0.96	0.120^§^
Food present	3.2 ± 0.94	3.3 ± 0.92	3.3 ± 0.93	0.034^§^
Food taste	3.4 ± 0.88	3.5 ± 0.90	3.5 ± 0.89	0.015^§^
**PSQI**	**7.8** **±3.75**	**8.4** **±3.30**	**8.2** **±3.48**	**0.007** ^**§**^
**Sleep quality**				
Good	142 (27.2)	170 (19.4)	312 (22.3)	0.001#
Poor	380 (72.8)	708 (80.6)	1,088 (77.7)	
**sQ-HPF total score**	**7.0** **±2.8**8	**6.8** **±2.78**	**6.9** **±2.82**	**0.101** ^**§**^
**Ultra processed food consumption**				
Low	164 (31.4)	298 (33.9)	462 (33.0)	0.332#
High	358 (68.6)	580 (66.1)	938 (67.0)	
**EEQ total score**	**10.5** **±5.60**	**11.3** **±5.98**	**11.0** **±5.85**	**0.019** ^**§**^
**EEQ sub-dimensions**				
Disinhibition	5.9 ± 3.84	6.1 ± 4.05	6.0 ± 3.97	0.291^§^
Type of food	2.8 ± 1.37	2.9 ± 1.37	2.8 ± 1.37	0.066^§^
Guilt	1.9 ± 1.36	2.3 ± 1.53	2.1 ± 1.48	< 0.001^§^

^*^FNL, Food and Nutrition Literacy; sQ-HPF, Screening Questionnaire for Highly Processed Food Consumption; PSQI, Pittsburgh Sleep Quality Index; UPF, Ultra-Processed Food; SD, Standard deviation.

Statistical significance was determined by Chi-square test (#) or Independent-samples *t* tests (§), and exact *p*-values are presented in the table. Statistical significance was set at *p* < 0.05.

**Figure 1:**
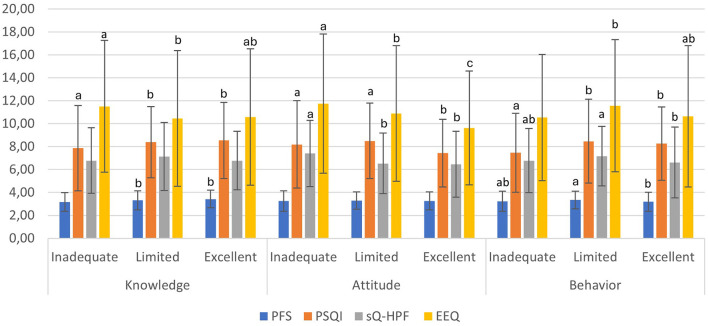
Comparison of PFS, PSQI, sQ-HPF, and EEQ scores across food and nutrition literacy levels. FNL, Food and Nutrition Literacy; sQ-HPF, Screening Questionnaire for Highly Processed Food Consumption; PSQI, Pittsburgh Sleep Quality Index; UPF, Ultra-Processed Food. One-way ANOVA was performed, followed by *post hoc* pairwise comparisons where appropriate (*p* < 0.05). Different letters indicate differences between groups (*p* < 0.05).

Figure 1 shows the comparison of hedonic hunger, sleep quality, UPF consumption, and emotional eating across FNL levels. In the knowledge sub-dimension of FNL, significant pairwise differences were observed for PFS and PSQI scores. *Post hoc* analyses indicated statistically significant differences between the inadequate and limited and inadequate and excellent groups for both outcomes, with lower PFS and PSQI scores observed in the inadequate group (*p* < 0.05). For EEQ, a statistically significant pairwise difference was observed only between the inadequate and limited groups, with higher EEQ scores in the inadequate group (*p* < 0.05). In contrast, sQ-HPF scores did not differ significantly across knowledge literacy levels (*p* > 0.05). In the attitude sub-dimension of FNL, no statistically significant differences were observed in PFS scores across literacy levels (*p* > 0.05). For PSQI, *post hoc* analyses indicated statistically significant pairwise differences between the excellent and inadequate and excellent and limited groups (*p* < 0.05), with lower PSQI scores observed in the excellent literacy group, indicating better sleep quality. With respect to sQ-HPF, statistically significant pairwise differences were observed between the inadequate and limited and inadequate and excellent groups (*p* < 0.05), with higher UPF consumption scores in the inadequate literacy group. For EEQ, statistically significant pairwise differences were identified across all literacy levels (*p* < 0.05), with the highest emotional eating scores observed in the inadequate group and the lowest scores in the excellent group. In the attitude sub-dimension of FNL, statistically significant pairwise differences were observed for PFS and sQ-HPF scores between the limited and excellent groups (*p* < 0.05). For PSQI, *post hoc* analyses indicated statistically significant pairwise differences between the inadequate and limited and inadequate and excellent groups (*p* < 0.05), with lower PSQI scores observed in the inadequate literacy group. Regarding EEQ, a statistically significant pairwise difference was identified between the inadequate and limited groups (*p* < 0.05), with lower emotional eating scores in the inadequate group.

The relationships between FNL sub-dimensions and age, anthropometric measures, hedonic hunger, sleep quality, UPF, and emotional eating are presented in Table 3. Age was significantly and negatively correlated with all FNL sub-dimensions, including knowledge (*r* = −0.161), attitude (*r* = −0.140), and behavior (*r* = −0.096); however, these associations were small in magnitude (all *p* < 0.001). Body weight and BMI were also negatively correlated with the knowledge (*r* = −0.164, *r* = −0.071, respectively) and attitude (*r* = −0.256, *r* = −0.154, respectively) sub-dimensions of FNL (*p* < 0.01), whereas no significant associations were observed with the behavior sub-dimension (*p* > 0.05). A positive and significant association was found between the PFS total score and both the knowledge (*r* = 0.113, *p* < 0.001) and attitude (*r* = 0.075, *p* = 0.005) sub-dimensions of FNL, while no significant relationship was detected with behavior. PSQI scores showed significant positive correlations with knowledge (*r* = 0.128, *p* < 0.001) and behavior (*r* = 0.103, *p* < 0.001), but not with attitude (*p* > 0.05). The sQ-HPF total score was negatively and significantly correlated with the attitude sub-dimension of FNL (*r* = −0.172, *p* < 0.001), whereas no significant associations were observed with knowledge or behavior. EEQ scores demonstrated significant negative correlations with the knowledge (*r* = −0.085, *p* = 0.001) and attitude (*r* = −0.128, *p* < 0.001) sub-dimensions, while their association with behavior was not statistically significant.

**Table 3:** Correlation between food and nutrition literacy and selected variables.

**Variables**	**FNL**					
	**Knowledge**		**Attitude**		**Behavior**	
	***r***	***p***	***r***	***p***	***r***	***p***
Age (y)	−0.161	< 0.001^**^	−0.140	< 0.001^**^	−0.096	< 0.001^**^
Body weight (kg)	−0.164	< 0.001^**^	−0.256	< 0.001^**^	0.008	0.756
BMI	−0.071	0.008^**^	−0.154	< 0.001^**^	−0.018	0.496
PFS total score	0.113	< 0.001^**^	0.075	0.005^**^	−0.050	0.063
PSQI	0.128	< 0.001^**^	−0.031	0.251	0.103	< 0.001^**^
sQ-HPF total score	0.024	0.366	−0.172	< 0.001^**^	−0.029	0.284
EEQ total score	−0.085	0.001^**^	−0.128	< 0.001^**^	0.023	0.384

FNL, Food and Nutrition Literacy; sQ-HPF, Screening Questionnaire for Highly Processed Food Consumption; PSQI, Pittsburgh Sleep Quality Index; UPF, Ultra-Processed Food.

^**^Correlation is significant at the 0.01 level. Spearman correlation coefficient.

The results of the multiple linear regression analyses examining factors associated with FNL sub-dimensions are presented in Table 4. When factors influencing the knowledge sub-dimension of FNL were analyzed using linear regression, the model was found to be statistically significant (R^2^ = 0.122; *p* < 0.001). Age (β = −0.142, *p* < 0.001) and gender (β = −0.241, *p* < 0.001) were negatively associated with knowledge scores. In contrast, higher scores on the PFS (β = 0.115, *p* < 0.001) and PSQI (β = 0.102, *p* < 0.001) were associated with higher knowledge scores. The EEQ total score was negatively associated with the knowledge sub-dimension ((β = −0.151, *p* < 0.001). BMI and sQ-HPF total score were not found to be significantly associated with knowledge scores (*p* >0.05). Similarly, when factors influencing the attitude sub-dimension were analyzed, the regression model was also statistically significant (R^2^ = 0.142; *p* < 0.001). Age (β = −0.117, *p* < 0.001), gender (β = −0.238, *p* < 0.001), sQ-HPF (β = −0.153, *p* < 0.001), and EEQ (β = −0.141, *p* < 0.001) were negatively associated with attitude scores. Higher PFS total scores were found to be positively associated with the attitude sub-dimension (β = 0.141, *p* < 0.001). BMI showed a borderline association with attitude scores (*p* = 0.050), while PSQI was not significantly associated with the attitude sub-dimension (*p* > 0.05). When the behavior sub-dimension of FNL was examined, the regression model was found to be statistically significant, although the explained variance was relatively low (R^2^ = 0.027; *p* < 0.001). It was determined that increasing age and poorer sleep quality (PSQI) were negatively associated with behavior scores (*p* < 0.001). Additionally, higher PFS total scores were associated with lower behavior scores (β = −0.078, *p* = 0.006). Gender, BMI, sQ-HPF, and EEQ were not significantly associated with the behavior sub-dimension (*p* > 0.05).

**Table 4:** Multiple linear regression analysis examining factors associated with food and nutrition literacy prediction.

**Variables**	**FNL**											
	**Knowledge**				**Attitude**				**Behavior**			
	**B (SE)**	**Beta**	***t***	***p***	**B (SE)**	**Beta**	***t***	***p***	**B (SE)**	**Beta**	***t***	***p***
Age	−0.223 (0.040)	−0.142	−5.622	< 0.001	−0.505 (0.108)	−0.117	−4.693	< 0.001	−0.370 (0.102)	−0.097	−3.631	< 0.001
Gender	−1.474 (0.160)	−0.241	−9.213	< 0.001	−3.993 (0.434)	−0.238	−9.193	< 0.001	0.241 (0.411)	0.016	0.587	0.557
BMI	0.006 (0.022)	0.007	0.259	0.796	−0.120 (0.061)	−0.053	−1.961	0.050	−0.038 (0.058)	−0.019	−0.658	0.511
PFS total score	0.415 (0.098)	0.115	4.234	< 0.001	1.400 (0.266)	0.141	5.258	< 0.001	−0.690 (0.252)	−0.078	−2.740	0.006
PSQI total score	0.087 (0.022)	0.102	3.986	< 0.001	−0.095 (0.059)	−0.041	−1.608	0.108	0.230 (0.056)	0.111	4.119	< 0.001
sQ-HPF total score	0.030 (0.027)	0.028	1.099	0.272	−0.440 (0.074)	−0.153	−5.981	< 0.001	−0.071 (0.070)	−0.028	−1.013	0.311
EEQ total score	−0.076 (0.014)	−0.151	−5.501	< 0.001	−0.196 (0.038)	−0.141	−5.200	< 0.001	0.058 (0.036)	0.047	1.614	0.107
	R^2^ = 0.122; *p* < 0.001				R^2^ = 0.142; *p* < 0.001				R^2^ = 0.027; *p* < 0.001			

Variable values: Gender (Male = 1, Female = 0).

## 4 Discussion

This study demonstrates that FNL is associated with hedonic hunger, emotional eating, sleep quality, and UPF consumption within the sample of university students. In addition, age and sex were found to be related to certain subdimensions of FNL. However, given the cross-sectional design of the study, these associations cannot be interpreted causally. The results suggest that eating behaviors among university students should be evaluated not only in relation to the knowledge component of FNL, but also by considering differences in attitudes and behaviors, together with psychobiological factors such as sleep patterns and emotional states. Moreover, it should be noted that UPF consumption in the present study was assessed using a screening-based instrument, which captures relative exposure patterns rather than detailed quantitative dietary intake. Therefore, the observed associations should be interpreted as indicative of UPF consumption tendencies rather than precise estimates of energy or nutrient intake. The university years represent a distinctive life stage during which individuals transition away from parental supervision and begin to manage their own food environments. During this period, limited culinary skills and budget constraints may render UPF consumption a common and accessible option for many university students (8). In one study reported that 42.1% of students ate outside at least 2–3 times per week, with most of these meals consisting of ultra-processed fast foods (6). In a study conducted in Türkiye in 2022, daily UPF consumption was most frequently reported for chocolate and wafers (19%), packaged bakery products (13.8%), and carbonated beverages (10.3%), while the proportion of students consuming chocolate and wafers three to five times per week reached 25.6% (32). Data from Japan further demonstrate that energy intake derived from UPFs decreases with increasing age and lower among adults aged 60–79 years compared with those aged 18–39 years (33). Similarly, studies conducted in Portugal and Canada have reported high levels of UPF consumption, particularly among children, adolescents, and young adults (34, 35). In the Canadian study, differences in UPF consumption according to sex, educational level, income adequacy, and region of residence were reported to be relatively limited (34). Consistent with these findings, the present study observed no statistically significant differences between sexes in terms of total sQ-HPF scores or low–high consumption categories. This lack of sex-based differences suggests that UPF consumption may be widespread among university-aged young adults, with gender distinctions being less pronounced in this population. Factors inherent to university life, such as academic demands, time constraints, and the need for convenience, may contribute to higher UPF consumption among both sexes (2, 8). Supporting this interpretation, a study conducted across five Mediterranean countries reported that more frequent eating out and higher number of daily meals were associated with an increased likelihood of unhealthy UPF consumption (36).

In addition, UPFs are often produced to be hyper-palatable through the use of synthetic flavor enhancers, and the the consumption of foods such as pizza, chips, and sugar-sweetened beverages has increasingly become normalized within many social contexts (3, 6). Furthermore, the inverse association observed between UPF consumption and the attitude dimension of FNL in both correlation and group comparison analyses suggests that attitude-related components such as valuing and prioritizing healthy eating, may be more closely related to UPF preferences than nutrition knowledge alone. However, given the observational nature of the study and the modest magnitude of some associations, these findings should be interpreted as indicative of relational patterns rather than causal mechanisms. Evidence from another study conducted in a Turkish sample has demonstrated associations between UPF consumption and psychological and behavioural determinants including hedonic hunger, food addiction, and mood-related symptoms (17), highlighting the multifactorial nature of UPF intake behaviours.

Enhancing FNL may contribute to more informed and reflective decision-making regarding eating behaviours (7). FNL is commonly conceptualized as comprising divided into two interrelated domains: cognitive literacy (knowledge and understanding) and practical literacy (skills and application); however, concordance between these domains is not always observed in real-life settings (12). In a study conducted with 765 students in Beijing, nutrition knowledge scores were relatively high, whereas food choice skills and food preparation competencies were significantly lower (12). This finding suggests that awareness of healthy eating principles does not necessarily translate into consistent behavioural implementation A similar pattern was observed in the present study. Female students demonstrated significantly higher scores than male students in the knowledge and attitude sub dimensions of FNL, while no significant difference was observed in the behaviour sub dimension. This discrepancy indicates that higher levels of nutrition-related knowledge and positive attitudes may not directly correspond to healthier eating behaviours The observed sex-related differences are consistent with previous evidence reporting higher levels of nutrition knowledge and health awareness among women (12, 37, 38), although the absence of behavioural differences underscores the importance of addressing structural, environmental, and psychosocial barriers that may constrain the translation of literacy into practice.

In the present study, the associations between UPF consumption and FNL were most evident within the attitude sub dimension. Higher UPF consumption observed among individuals with lower attitude scores suggests that positive orientations toward nutrition such as valuing and prioritizing healthy eating may be more closely related to UPF avoidance than nutrition knowledge alone. The absence of a significant difference in the knowledge sub dimension further indicates that UPF consumption cannot be attributed solely to insufficient nutritional knowledge. Rather, a range of contextual and behavioural factors, including taste preferences, accessibility, cost, and time constraints are likely to influence food choices among university students. Among that attributed solely economic constraints appear particularly important. The relatively high cost of fresh fruits and vegetables compared with the low price and long shelf life of UPFs may discourage healthier choices among students with limited financial resources (6, 39). In this context, the potential protective role of FNL may be attenuated by food insecurity. It has been reported that, approximately 41% of university students experience some degree of food insecurity, a condition that may prompt the selection of inexpensive and energy-dense foods for economic reasons, even among individuals who are aware of dietary recommendations (40). Evidence from previous studies supports the notion that the relationship between FNL and UPF consumption may vary across population subgroups. In a study involving 7,761 adolescents in China, an inverse association between FNL and UPF consumption scores was observed among girls but not among boys (7). Other research indicates that students with higher nutrition literacy may adopt more adaptive eating strategies during academically demanding periods, such as examination weeks, by preferring foods that support cognitive performance (e.g., nuts, fruits, and whole grains) over energy drinks and sugary snacks (12). From a conceptual perspective, FNL may function as a mediating factor between environmental influences such as marketing exposure and easy access to low-cost UPFs and individual consumption behaviours (14). For example, even in campus settings where access to healthy foods is limited, students with higher literacy levels may employ compensatory strategies, such as selecting the least processed options available or preparing meals at home. A study conducted among adults in Türkiye, identified statistically significant differences between individuals with low and high UPF consumption with respect to age, sex, marital status, cooking, and food preparation skills, and overall food literacy scores (2). F Collectively, these findings suggest that while FNL may play a protective role within the contemporary food environment, this role is likely to be contingent upon not only knowledge and attitudes but also practical skills and broader economic and support mechanisms.

When evaluated hedonic hunger, the finding that women exhibited higher total PFS scores, as well as higher scores on specific sub dimensions (food availability and food taste), suggests a greater sensitivity to emotional and environmental eating cues within the study sample. Similarly, women demonstrated higher total EEQ scores compared with men. Analysis of the EEQ sub dimensions indicated that guilt scores were significantly higher among women, whereas no significant sex differences were observed in the disinhibition and food type sub dimensions. These findings are consistent with previous literature indicating that hedonic hunger is commonly associated with stress, emotional fluctuations, and sleep disturbance, factors that are frequently reported among university students (41, 42). However, given the cross-sectional nature of the data, these relationships should be interpreted as associative rather than causal, and the practical relevance of statistically significant differences with modest effect sizes should be considered cautiously.

In the present study PFS scores were positively associated with the knowledge and attitude sub dimensions of FNL, but a negatively associated with the behaviour sub dimension. The positive association observed between hedonic hunger and the knowledge and attitude sub dimensions of FNL may reflect a dissociation between cognitive awareness and behavioural self-regulation. Higher levels of nutrition-related knowledge and favourable attitudes do not necessarily translate into effective control over hedonically driven eating behaviours, particularly in food environments characterized by heightened reward sensitivity and extensive exposure to UPFs. At the behavioural level, the implementation of healthy practices may be constrained by factors such as self-regulation, routines, time and economic constraints, and sleep-related challenges. Previous research has indicated that the relationship between hedonic hunger and UPF consumption may differ according to individual characteristics, including levels of self-control and impulsivity (43). Hedonic hunger is commonly defined as the motivation to eat driven by the anticipated pleasure and reward value of food, independent of biological energy homeostasis (44). This tendency may become particularly pronounced in food environments characterized by the widespread availability of UPFs that are designed to stimulate reward pathways (45, 46). UPFs are characterized by combinations of high fat and refined sugars that are rarely found together in natural foods. This combination may enhance the palatability and reward value of foods by engaging dopaminergic pathways within the mesolimbic reward system, eliciting neural responses that resemble those observed with substances associated with addictive-like eating behaviours (47). In addition, unlike minimally processed foods, the absence of a fibrous matrix in many UPFs is associated with faster gastrointestinal digestion and absorption, leading to more rapid postprandial glycaemic responses. Such rapid absorption has been proposed to attenuate satiety-related hormonal signalling (e.g., glucagon-like peptide-1 and peptide YY) while promoting appetite-stimulating signals such as ghrelin, thereby potentially interfering with homeostatic appetite regulation (44). Nevertheless, these mechanisms are primarily derived from experimental and mechanistic studies and should be interpreted with caution when extrapolated to observational findings.

University students may be particularly vulnerable to emotional eating, which is often described as a maladaptive coping strategy and has been linked to sleep disturbances and mental health challenges (48). In the present study, the inverse associations observed between emotional eating levels and the knowledge and attitude sub dimensions of FNL suggest that psychological eating behaviours may be accompanied by reduced engagement with healthy eating principles. However, these findings do not imply directionality and should be interpreted within the limits of the cross-sectional design. Moreover, a growing body of evidence has reported associations between higher UPF intake and adverse mental health indicators, including depressive symptoms and anxiety (16, 18, 49). These findings underscore the complex interplay between psychological well being, eating behaviours, and food environments, rather than indicating a direct causal pathway.

In a study conducted among university students in Türkiye, higher UPF consumption was associated with poorer physical quality of life and greater psychological distress (50). Similarly, a study from Brazil reported that younger age and higher perceived stress levels were related to increased UPF consumption (51). Among young individuals (age < 40 years), higher levels of UPF consumption have been found to be significantly associated with depressive symptoms compared with lower consumption levels (49). In another study conducted in Türkiye, UPF consumption was positively associated with food addiction and psychological symptoms such as depression, anxiety, and stress (17). These findings indicate a potential relationship between UPF consumption and mental health outcomes. However, the underlying pathways remain unclear. It has been suggested that this relationship may involve disruptions in physiological eating regulation (e.g., heightened food anticipation or binge-eating tendencies) and/or inflammatory processes linked to nutritional factors such as high sugar and unhealthy fat content, as well as non-nutritional components including food additives (49). Given the observational nature of much of the available evidence, further longitudinal and experimental studies are warranted to clarify these mechanisms. Accordingly, strategies aimed at reducing UPF consumption may be considered as potential approaches to support both physical and mental well being, although causal effects cannot be inferred from the present findings.

In the present study, higher PSQI scores observed among female students suggest that sleep disturbances may be more prevalent among university-aged women within the study sample. In addition, the association between lower levels of FNL and poorer sleep quality suggests that FNL may influence not only to dietary choices but also broader lifestyle behaviours. Previous studies have reported that short sleep duration and poor sleep quality are associated with a greater tendency toward UPFs consumption and with alterations in appetite-regulating hormones (52, 53). Consistent with these findings, a study conducted in Southern Italy reported that individuals in the highest quintile of UPF consumption had a higher likelihood of poor sleep quality compared with those in the lowest consumption group (49). Within this context, impaired sleep quality may indirectly contribute to UPF preferences by intensifying hedonic hunger and reinforcing emotional eating behaviours. Similarly, Ateş et al. reported that the relationships between hedonic hunger, sleep quality, and mental well being may be particularly significant among young adults (41). In the present study, the association of observed between PSQI scores and FNL sub dimensions, particularly the inverse association with the behaviour sub dimension identified in regression analyses, suggest the presence of a sleep–eating behaviour cycle, although the directionality of this relationship cannot be determined.

The relationship between sleep and nutrition is increasingly conceptualized as bidirectional and potentially self-reinforcing, rather than unidirectional. Sleep deprivation may be associated with reduced decreases activity in the prefrontal cortex, which is involved in executive function and self-regulation, alongside increased responsivity in reward-related brain regions such as the amygdala (54, 55). Within this framework, insufficient sleep has been proposed to heighten sensitivity to rewarding food cues, which may be reflected in a greater preference for sugar- and fat-rich UPFs on subsequent days. While the tryptophan–melatonin pathway illustrates one mechanism through which dietary factors may influence sleep, sleep deprivation itself has been shown to disrupt appetite regulation via hormonal alterations, including reduced leptin and elevated ghrelin levels (54). In this context, the finding that PSQI scores significantly predicted the behaviour sub dimension of FNL in the present study suggests that the translation of FNL and attitudes into actual behaviours may be more closely related to sleep quality. However, given the cross-sectional design in this study, this association should be interpreted cautiously. From public health perspectives, these findings support the potential value of considering sleep hygiene as a complementary component of FNL interventions targeting university students, rather than as a direct outcome of such programs.

Beyond individual-level factors, the broader food environment plays a critical role in shaping dietary behaviours. The obesity epidemic has been linked to obesogenic food environment that promotes UPF consumption through high palatability, low nutritional quality, high energy density, affordability, and convenience. In a study conducted among adults with obesity in Southern Italy, higher UPF consumption was observed alongside increasing BMI, accompanied by reduced adherence to the Mediterranean Diet (56). In the present study, body weight and BMI were negatively associated with the knowledge and attitude sub dimensions of FNL, while no significant association was observed with the behaviour sub dimension. This pattern suggests that university students with higher body weight may demonstrate lower nutrition-related knowledge and less favourable dietary attitudes; however, these factors alone do not appear sufficient to explain dietary behaviours. The observed dissociation between knowledge and attitude vs. actual behaviour may reflect the dominant influence of environmental and structural determinants, such as the widespread availability of UPFs, their relatively low cost, and the time-saving advantages they offer. Accordingly, these findings indicate that the relationship between UPF consumption and obesity cannot be explained solely by individual knowledge deficits. Rather, attitudes, environmental factors, and the structural characteristics of the contemporary food environment should be considered jointly when interpreting dietary behaviours in university student populations. The interventions aimed at improving FNL should not be limited solely to information transfer; rather, comprehensive programs integrating components such as emotional regulation and sleep quality may be more effective. An intervention study conducted among first-year University students reported that an education program based on conscious nutrition and mindful eating was able to improve FNL and eating awareness, highlighting the feasibility of campus-based programs (57).

This study also has several methodological and conceptual strengths. The large sample size increases the statistical power. And supports more stable estimates in multivariate analyses, including multiple linear regression models. This feature represents an important advantage for examining complex relationships among multiple variables within a university student sample. In addition, the concurrent assessment of psychobiological factors related to eating behaviours constitutes a notable strength. Examining hedonic hunger, emotional eating, and sleep quality within the same analytical framework, the study provides a more integrated perspective, suggesting that eating behaviours among university students are shaped not only by cognitive aspects of literacy but also by emotional and physiological processes. While these strengths enhance the interpretability of the findings, they should be considered alongside the study's observational design when drawing broader inferences.

However, this study also has several limitations, and the findings should be interpreted within this context. First, the cross-sectional design of the study does not allow the relationships between FNL and UPF consumption, hedonic hunger, emotional eating, and sleep status to be interpreted within a causal framework. Consequently, the observed associations should be considered relational rather than indicative of directionality. Second, the assessment of key variables such as FNL, UPF consumption, sleep quality, and eating behaviours was based on self-reported data, which may be subject to recall bias, social desirability bias, and the possibility of misreporting. Moreover, body weight and height were self-reported, which may have led to systematic underestimation of body weight and BMI. Therefore, associations involving BMI should be interpreted with caution. In the present study, BMI was included in the regression models as a covariate to control for potential confounding rather than as a primary exposure variable. In addition, the use of snowball sampling in the present study may have introduced selection bias. This approach may limit the representativeness of the sample with respect to the broader university student population and, consequently, reduce the generalizability of the findings. Therefore, the results should be interpreted within the context of university students with similar sociodemographic characteristics. Third, UPF consumption was evaluated using a screening-based scale which does not provide detailed quantitative information on dietary intake, such as total energy intake or macro- and micronutrient composition. Fourth, sleep status was assessed solely using the PSQI, and objective sleep measures were not included in the study. In addition, some potential confounding variables that may influence eating behaviours and sleep pattern such as physical activity level, academic stress, and socioeconomic status, caffeine or energy drink consumption were not assessed. The absence of these factors may have affected the strength of the observed relationships and may partly account for the relatively low explained variance in certain regression models, especially for the behaviour sub dimension. Furthermore, as the study sample consisted entirely of university students within a specific age range, the generalizability of the findings to the general population, other age groups, or young adults outside the university setting is limited. Finally, although several associations reached statistical significance, the observed effect sizes were generally small. This suggests that statistical significance, likely influenced by the large sample size, does not necessarily imply strong practical or clinical relevance. The findings should therefore be interpreted as indicating modest associations rather than large effects. Nevertheless, even small effects may be meaningful at the population level, particularly when multiple behavioural and psychosocial factors interact to influence eating behaviours and sleep quality among university students.

## 5 Conclusions

The results of this study indicate that FNL among university students is associated with hedonic hunger, emotional eating, sleep quality, and UPF consumption. Higher levels of FNL in the knowledge and attitude domains were associated with lower UPF consumption, better sleep quality, and less pronounced emotional eating behaviors, whereas lower literacy levels were related to higher hedonic hunger, poorer sleep quality, and more maladaptive eating patterns. The findings also suggest that age and sex play are relevant factors in shaping FNL. Although UPF consumption did not differ significantly by sex, its inverse association with the attitude dimension of FNL underscores the importance of psychosocial and behavioral determinants beyond demographic characteristics alone. These findings highlight the multifaceted nature of eating behaviors among university students and suggest that literacy related factors interact with psychological and lifestyle components.

From a public health perspective, university-based nutrition education programs that holistically integrated behavioral and psychological components are needed. Policies and interventions targeting young adults may benefit from prioritizing the promotion of sustainable healthy eating behaviors, addressing the pervasive influence of UPFs within food environments, and fostering settings that support both physical and mental well being. Future research should explore integrated educational approaches that combine nutrition education with media literacy, emotional regulation strategies, and practical food preparation skills. In addition, adopting healthier campus food environments such as increasing access to fresh foods and encouraging healthier food choices may represent a complementary strategy, although the effectiveness of such measures requires evaluation through longitudinal and intervention studies. From a clinical standpoint, a more holistic assessment of student health that considers both dietary habits and sleep quality may be warranted. In conclusion, the university period represents a critical window of opportunity during which lifelong dietary habits are shaped and improving FNL during this stage may constitute a promising, albeit context-dependent, avenue for supporting healthier trajectories into adulthood.

## Data Availability

The data presented in this study are available on request from the corresponding author due to ethical and privacy restrictions.
